# Age-Adjusted D-Dimer in the Prediction of Pulmonary Embolism: Does a Normal Age-Adjusted D-Dimer Rule Out PE?

**DOI:** 10.1155/2017/4867060

**Published:** 2017-10-19

**Authors:** Jacob Ortiz, Rabia Saeed, Christopher Little, Saul Schaefer

**Affiliations:** ^1^Department of Internal Medicine, Division of Cardiovascular Medicine, University of California Davis, Davis, CA, USA; ^2^Cardiology Section, Department of Veteran Affairs, Northern California Health Care System, Mather, CA, USA

## Abstract

Risk assessment for pulmonary embolism (PE) currently relies on physician judgment, clinical decision rules (CDR), and D-dimer testing. There is still controversy regarding the role of D-dimer testing in low or intermediate risk patients. The objective of the study was to define the role of clinical decision rules and D-dimer testing in patients suspected of having a PE. Records of 894 patients referred for computed tomography pulmonary angiography (CTPA) at a University medical center were analyzed. The clinical decision rules overall had an ROC of approximately 0.70, while signs of DVT had the highest ROC (0.80). A low probability CDR coupled with a negative age-adjusted D-dimer largely excluded PE. The negative predictive value (NPV) of an intermediate CDR was 86–89%, while the addition of a negative D-dimer resulted in NPVs of 94%. Thus, in patients suspected of having a PE, a low or intermediate CDR does not exclude PE; however, in patients with an intermediate CDR, a normal age-adjusted D-dimer increases the NPV.

## 1. Introduction

Diagnosis of pulmonary embolisms (PE) continues to challenge physicians largely because of the wide spectrum of presentations from vague symptoms to profound illness [1]. Clinical decision rules (CDR), such as the Wells rule [2] and Geneva score [3], have classically been employed to risk, assess, and triage patients for diagnostic imaging for PE. Derived from cohort studies and prospectively validated, these two CDR are often lauded for their high negative predictive value (NPV) in the low risk groups (Wells rule 99.5%, Geneva score 91.0%). However, a major criticism is that these CDR score too many patients as intermediate risk, where the predictive value diminishes and thus promotes an overuse of CT imaging when implemented in clinical practice. Previous efforts to refine these scores—modified Wells score and simplified revised Geneva—have not resolved this issue, showing again strong performance in low risk groups but marginal performance in those with higher scores [4].

Multiple studies have shown that combining D-dimer testing with either CDR improves their overall negative predictive value (NPV) and positive predictive value (PPV) [5]. Le Gal and Wells have opined [6] that “a negative D-dimer assay safely rules out the diagnosis of PE in patients with a low-intermediate or unlikely clinical probability,” while a retrospective trial by Van Es et al. [7] demonstrated that using D-dimer cutoffs less than 500 ug/L would effectively exclude PE (NPV 0.988). Conversely, a D-dimer greater 1000 ug/L suggested PE and referral for CTPA [7]. These data have been refined by the recent* ADJUST-PE* trial, which showed that PE could be safely excluded in a larger number of patients using an age-adjusted cutoff for D-dimer (age ×10 ug/L with a lower limit of 500 ug/L) [8]. Similarly, using high-value elements of Wells rule, hemoptysis, signs of deep vein thrombosis, and “PE most likely,” was shown to add incremental value to the D-dimer test [7]. Keeping a high NPV, a larger percentage of the patients (36%) was able to be excluded without CTPA; however, the predictive value of an intermediate score remained low [9].

Thus, the exact role of D-dimer testing in the cohort of patients with an intermediate probability CDR remains still in question. In order to help clarify this issue, we performed a retrospective analysis of patients referred for CTPA to (a) confirm the predictive value of the Wells rule, Geneva score, and their individual elements in a University hospital population referred for CTPA to diagnose or rule out PE and (b) determine the utility of D-dimer testing in the population of patients with low and intermediate CDR scores.

## 2. Methods

This study was approved by the Institutional Review Board of the University of California Davis. Imaging reports of all patients referred for CTPA to evaluate for PE between 2012 and 2015 (*n* = 4756) were screened for inclusion/exclusion criteria and 894 charts were reviewed by the authors. Patients were referred by either an emergency room physician or a hospital inpatient physician. Charts were reviewed for individual variables of the both CDR, D-dimer, and other commonly referenced signs of PE, which were analyzed in a database after subtracting all patient identifiers.

Data Collection: An encrypted database was constructed to store all clinical variables. Each chart was reviewed independently by two medical researchers who pulled real-time data at the onset of symptoms. Heart rate, pulse oximetry, and chief complaint were selected from either triage nursing or floor nursing assessment. Key past medical history, including previous thromboembolic disease, active cancer, recent surgery, or immobility, was added from history and physical note during that admission. Clinical assessment measures, presence of unilateral lower extremity edema, tenderness, and cardiopulmonary exam were collected from notes during that evaluation. D-dimer (immunoturbidimetric assay, Instrumentation Laboratory, Bedford, MA) and chest imaging was collected from stored diagnostics from that admission. Blood gas data were employed only if results were collected within 4 hours from onset of symptoms. The original Wells rule [10] and revised Geneva score [3] were determined using the same medical calculator.

### 2.1. Statistical Analysis

Logistic regression was used to model the probability of PE by each score, score element, and other clinical characteristic. Sensitivity, specificity, negative predictive value, and positive predictive value were calculated from 2 × 2 tables of predicted and actual outcomes. Binomial confidence intervals were calculated using the Agresti-Coull method [11]. Data are presented as mean +/− standard deviation. Significance was defined as a *P* value < 0.05.

## 3. Results

### 3.1. Predictive Value of Wells Rule and Geneva Score

The final cohort assembled in this study included patients spanning an age range of 18–98, mean age 55.4, with a representative diverse population (Table 1). The overall incidence of pulmonary embolism was 15.3%, similar to that seen in a large meta-analysis (14%) [9]. The incidence of a CTPA positive for PE of each stratification from Wells rule was similar to the original papers (e.g., 5.6% for low risk in the current study versus 5%) [2], but there was considerable variation between the incidence of PE in this cohort for intermediate and high risk groups via revised Geneva score compared to that original paper [3] (e.g., 41.3% in our study versus 74%).

The Wells rule and revised Geneva score, as well as individual components of both CDR and signs of poor oxygenation, were analyzed and depicted on receiver operating characteristic (ROC) curves (sensitivity against 1 – specificity). The predictive power of the Wells rule and Geneva score are shown in Tables 2 and 3, respectively. In each CDR, only clinical signs of DVT and history of previous DVT were significantly different between the patients with and without PE. Signs of DVT were superior to all individual elements and both CDR with an area under the curve (AUC) of 0.80, although most patients with a positive CTPA did not have evidence of DVT (34% PPV and 89% NPV). The Wells rule overall had an area under the curve (AUC) of 0.70 compared to the AUC of 0.69 found with the revised Geneva score, while no individual elements of either CDR, such as age, tachycardia, symptoms of pleuritic chest pain, surgery, and immobilization/surgery, had an AUC > 0.55. Evaluation of the variable infamously known as “PE most likely” has been challenging to successfully assess but, nebulous as it is, was shown to be fairly predictive in prior studies [9]. Using a more rigorously defined protocol to identify chronic obstructive pulmonary disease (COPD) exacerbation, pneumonia, acute coronary syndrome (ACS), or congestive heart failure (CHF) as dispositive of “PE most likely” yielded an AUC of 0.63.

### 3.2. D-Dimer

Subgroup analysis of patients whose D-dimer was measured (*n* = 173) showed that-adjusted D-dimer (age ×10 ug/L with a lower limit of 500 ug/L) [8] had a NPV of 0.96 and PPV of 0.32 alone and independent of CDR. The average age-adjusted D-dimer in patients with a positive CTPA was significantly greater than those patients with a negative CTPA (4645 +/− 7440 ug/L, compared to 385 +/− 82 ug/L, mean +/− SD, *P* < 0.001). There was no correlation between age-adjusted D-dimer and either the Wells rule or Geneva score (*r* = 0.25 and 0.28, resp.). Overall, the addition of D-dimer to high-value elements did not incrementally increase the predictive power (e.g., signs of DVT plus D-dimer had an AUC of 0.79 compared to 0.80 and 0.78 of these items alone).

### 3.3. D-Dimer in Low and Intermediate Risk Patients

The distribution of positive and negative CTPA and D-dimer by CDR risk is shown in Table 4. As the population with low-to-intermediate risk by CDR is most problematic, further analysis examined the discriminatory ability of coupling D-dimer values with low or intermediate Wells rule or Geneva score to improve their predictive value. Excluding high risk scores from the cohort left a subset of patients with low or intermediate scores (*n* = 678 according to Wells and *n* = 841 according to Geneva).

In the patients with a* low* CDR indicated by Wells rule <2, overall 166 of 176 (94.4%) patients had a negative CTPA (Table 4). A low risk Wells rule with a positive D-dimer yielded 2 patients with a positive CTPA. However, combining a low risk Wells rule with a negative D-dimer did not result in any positive CTPA studies (NPV 100%). Similarly, a low risk Geneva score alone resulted in a 94.4% negative CTPA rate (149/158), while the addition of a negative D-dimer effectively ruled out PE; that is, no patients with a low risk Geneva score and a negative D-dimer had a positive CTPA. Thus, the combination of a low risk Wells rule or Geneva score, coupled with a negative D-dimer, resulted in no patients with a positive CTPA.

With either CDR,* intermediate* scores (Wells 2–6 or Geneva score 4–10, Table 5(b)) alone had a negative CTPA rate of 89.7% and 86.1%, respectively. Subgroup analysis of those with a measured D-dimer identified 119 patients with an intermediate Wells rule or Geneva score. Of the 16 patients with an intermediate Wells score and a +CTPA, 12 (75%) had an elevated age-adjusted D-dimer. Conversely, only 4 of the 68 (5.8%) patients with an intermediate Wells value and a negative age-adjusted D-dimer had a positive CTPA (chi-square = 5.87, *P* = 0.02), yielding a NPV of 94.3%. In those patients with an intermediate Geneva score (119 patients scoring 4–10 on the CDR with a measured D-dimer), only 4 of the 67 (6.0%) patients with a negative age-adjusted D-dimer had a positive CTPA, with a similar NPV of 94.1%. Thus, the addition of age-adjusted D-dimer increased the NPV of an intermediate value from either CDR alone by ~6%.

## 4. Discussion

This study has several key findings: First, this analysis validates the modest performance of the Wells rule and Geneva score in predicting pulmonary embolism by CTPA as seen in the original studies [3, 10] and subsequent meta-analyses [7]. Within these CDR, signs of DVT were most predictive, but using firm criteria to exclude confounding alternative diagnoses did not significantly improve the ROC. Second, these data test the importance of D-dimer testing in stratifying patients for CTPA [9], finding that the age-adjusted D-dimer does not improve the NPV of the Wells and Geneva CDR in low scoring patients but does improve the NPV in intermediate scoring patients.

### 4.1. Wells Rule and Geneva Score

Recently, several studies have attempted to enhance CDR guiding PE diagnostic workup; these studies have isolated high-value elements such as signs of DVT [9] and expanded use of D-dimer [12, 13]. While this study found similar high-value elements (alternative diagnosis and signs of DVT), neither added value to the predictive power of either CDR. Signs of DVT, as expected, proved to be the most predictive clinical according to the ROC of 0.80, greater than either CDR. The argument could be made that this clinical finding is merely an indicator for venous thromboembolism rather than an element in a collective clinical predictive rule.

A unique feature of this paper is effort to remove the vague item “PE as the most likely diagnosis” by creating firm clinical criteria that could be uniformly applied. This method was slightly less predictive (0.63 AUC versus 0.70 in Van Es et al. [14]) compared to other studies, which suggests our method lacks the inherent clinical judgment in “PE as the most likely diagnosis” that predicts pulmonary embolism. A well-done study by Penaloza et al. [15] analyzed experienced physicians compared to strict regimented CDR studied here, showing a clear superiority of unstructured clinician gestalt compared to both CDR (AUC of 0.89 (95% CI 0.87 to 0.92) for gestalt, 0.76 (95% CI 0.72 to 0.79) for Wells score, and 0.72 (95% CI 0.68 to 0.76) for revised Geneva score). Rather than yielding that CDR will never equate to clinical experience, the Penaloza et al. paper may actually be highlighting the true predictive power of clinician experience.

In comparison with prior studies [3], fewer patients with an intermediate or high risk Geneva score in the current study had imaging evidence of a PE. This difference may be due to the prevalence of PE in the population, but is more likely the result of other investigators using an algorithm with sequential assessment of D-dimer in all patients and venous ultrasound in the vast majority of patients prior to CTPA [3]. As the current study was retrospective, the use of CTPA was often based on clinical judgment rather than sequential testing. This implies that the PPV of an intermediate or high Geneva score is improved with the consistent use of an algorithm.

### 4.2. D-Dimer in Low and Intermediate Risk Patients

The NPV of a low Wells rule is approximately 94%, and the addition of a negative D-dimer test effectively excludes PE. The NPV of an intermediate Wells or Geneva score alone was less than that of a low score (89% and 86%, resp.), while the addition of a negative age-adjusted D-dimer improved the NPV similarly to that of a low score (~94%). Likely due to the small number of patients with D-dimer testing, this increase was not statistically significant, with overlapping 95% confidence intervals. Importantly, a negative age-adjusted D-dimer did not rule out a +CTPA, as, overall, 4 patients with a negative age-adjusted D-dimer had a +CTPA (all in the intermediate risk group). Several studies have examined the role of D-dimer testing in improving the predictive value of a low or intermediate CDR. For example, Wells [6] recently stated that a negative D-dimer with a low-to-intermediate probability CDR “rules out” a PE, while Harringa et al. [13] and Gupta et al. [16] found that PE could be safely excluded with a NPV = 100% in patients with a non-high risk CDR and negative D-dimer. Similarly, Sohne et al. [17] found a 100% NPV in outpatients using this strategy. In contrast, there are case reports of patients with acute pulmonary embolism who are missed by the combination of a Wells PE unlikely score and a negative age-adjusted D-dimer [18], while Sohne et al. [19] did not find this strategy reliable to exclude PE in inpatients. Thus, while a low risk CDR coupled with a negative D-dimer effectively rules out PE, patients with a negative D-dimer and an intermediate Wells or Geneva score should likely proceed to further testing.

### 4.3. Limitations

This retrospective study did not prospectively enroll patients. However, all patients who had a CTPA over the enrollment period were included in the analysis, yielding a robust sample size that was diverse in age, gender, and race. In addition, only a subset of patients received D-dimer testing, limiting the conclusions regarding the utility of D-dimer testing. Finally, as the patients receiving D-dimer testing were selected by the diagnosing physician, they may have had different perceived risks for PE not reflected in the data. Thus, the conclusions may not be broadly applicable to all patients presenting with possible PE.

## 5. Conclusions

This study confirms the modest PPV and NPV of the Wells rule and Geneva score but affirms the value of clinical judgment (“PE most likely”) and the presence of signs of DVT as predictors of PE as diagnosed by CTPA. A normal age-adjusted D-dimer coupled with a low risk CDR effectively ruled out PE, while a negative D-dimer increased the NPV of an intermediate CDR to approximately 94%. Thus, the value of D-dimer testing, vis-à-vis clinical judgment, should be carefully evaluated in light of these findings and should not be used to “rule out” PE.

## Figures and Tables

**Table 1:** Characteristics of the study population.

Patient characteristic	No PE (*n* = 768)	PE (*n* = 126)
Age (years)	55.5 (17.4)	58.8 (14.7)
Gender (*n*, %)		
Female	440 (57.3%)	58 (46%)
Male	328 (42.7%)	68 (54%)
SpO_2_ (mean +/− SD)	95.4 (4.9)	94.5 (4.9)
PaO_s_	92.4 (51.7)	89.9 (52.4)
PaCO_2_	40.2 (12.1)	39.8 (12.1)
A-a gradient	117.2 (145.5)	132.2 (111.1)
D-Dimer	1066.7 (2121.7)	4029 (7037.1)
Wells rule	3.8 (2.1)	5.6 (2.5)
Geneva score	5.8 (2.6)	7.9 (3.4)
Symptom (*n*, %)		
CP	336 (43.7%)	41 (32.5%)
SOB	389 (50.6%)	80 (63.5%)
Unknown	36 (4.7%)	5 (4%)
Stasis (*n*, %)		
−	499 (65.0%)	77 (61.1%)
+	262 (34.1%)	49 (38.9%)
Surgery/fracture (*n*, %)		
−	683 (88.9%)	114 (90.5%)
+	68 (8.9%)	12 (9.5%)
Signs of DVT (*n*, %)		
−	681 (88.6%)	83 (65.9%)
+	80 (10.4%)	43 (34.1%)
Hemoptysis (*n*, %)		
−	737 (95.9%)	120 (95.2%)
+	24 (3.1%)	6 (4.8%)
Cancer (*n*, %)		
−	554 (72.1%)	82 (65.1%)
+	205 (26.6%)	44 (34.9%)
Unilateral limb pain (*n*, %)		
−	719 (93.6%)	97 (77%)
+	42 (5.5%)	29 (23%)
Previous DVT/PE (*n*, %)		
−	664 (86.4%)	97 (77%)
+	97 (12.6%)	29 (23%)
PNA (WBC CXR) (*n*, %)		
−	610 (79.4%)	109 (86.5%)
+	151 (19.7%)	17 (13.5%)
COPD (PCO2 Wheeze) (*n*, %)		
−	677 (88.1%)	116 (92.1%)
+	83 (10.8%)	10 (7.9%)
ACS/HF (*n*, %)		
−	641 (83.5%)	110 (87.3%)
+	120 (15.6%)	16 (12.7%)
Other diagnosis (*n*, %)		
−	716 (93.2%)	122 (96.8%)
+	34 (4.4%)	3 (2.4%)
Heart rate (*n*, %)		
≤100	366 (47.6%)	55 (43.7%)
>100	392 (51%)	71 (56.3%)
SpO2		
Under 75	96 (12.5%)	11 (8.7%)
75–94	199 (25.9%)	33 (26.2%)
95 or Above	463 (60.8%)	82 (65.1%)

CP: chest pain; SOB: shortness of breath; PNA: pneumonia; COPD: chronic obstructive pulmonary disease; ACS/HF: acute coronary syndrome/heart failure.

**Table 2:** Predictive power of Wells score and its elements.

	Data-defined cutoff^*∗*^	Odds ratio^†^ (95% CI)	*P* value^†^	Sensitivity^†^	Specificity^†^	Sensitivity + specificity
Wells score (continuous)	≥5	4.07 (2.76, 6.05)	<0.001	0.61	0.72	1.33
Wells Score > 4	—	3.11 (2.08, 4.75)	<0.001	0.71	0.55	1.27
HR > 100	—	1.21 (0.83, 1.77)	0.335	0.56	0.48	1.05
Stasis (+ versus −)	—	1.21 (0.82, 1.78)	0.332	0.39	0.66	1.04
Signs of DVT (+ versus −)	—	4.41 (2.84, 6.80)	<0.001	0.34	0.89	1.24
Hemoptysis (+ versus −)	—	1.54 (0.56, 3.61)	0.358	0.05	0.97	1.02
Cancer (+ versus −)	—	1.45 (0.97, 2.2)	0.069	0.35	0.73	1.08
Previous DVT/PE (+ versus −)	—	2.05 (1.27, 3.23)	0.003	0.23	0.87	1.10

^*∗*^Value at which probability of PE predicted by logistic regression model equals or first exceeds observed frequency of PE. ^†^At data-defined cutoff, where applicable.

**Table 3:** Predictive power of Geneva score and its elements.

	Data-defined cutoff^*∗*^	Odds ratio^†^ (95% CI)	*P* value^†^	Sensitivity^†^	Specificity^†^	Sensitivity + specificity
Geneva score (continuous)	≥7	2.84 (1.93, 4.24)	<0.001	0.64	0.61	1.26
HR > 74	—	1.52 (0.82, 3.08)	0.213	0.91	0.13	1.04
HR ≥ 95	—	1.19 (0.80, 1.77)	0.393	0.65	0.39	1.04
Stasis (+ versus −)	—	1.21 (0.82, 1.78)	0.332	0.39	0.66	1.04
Signs of DVT (+versus −)	—	4.41 (2.84, 6.80)	<0.001	0.34	0.89	1.24
Hemoptysis (+versus −)	—	1.54 (0.56, 3.61)	0.358	0.05	0.97	1.02
Cancer (+versus −)	—	1.45 (0.97, 2.15)	0.069	0.35	0.73	1.08
Unilateral Limb Pain (+versus −)	—	5.12 (3.03, 8.57)	<0.001	0.23	0.94	1.17
Previous DVT/PE (+versus −)	—	2.05 (1.27, 3.23)	0.003	0.23	0.87	1.10

^*∗*^Value at which probability of PE predicted by logistic regression model equals or first exceeds observed frequency of PE. ^†^For predictor categorized using data-defined cutoff, where applicable.

**Table 4:** Distribution of CTPA and D-dimer results by Wells rule and Geneva score.

CDR	Study population	CTPA			D-dimer		
		Patients (*N*=)	Pos (%)	Neg (%)	*N* = 173	Pos (%)	Neg (%)
Low risk	Wells rule (<2)	176	10 (5.6%)	166 (94.4%)	32	9 (28.1%)	23 (71.9%)
	Geneva score (<4)	158	9 (5.6%)	149 (94.4%)	35	8 (22.9%)	27 (77.1%)
Intermediate risk	Wells rule (2–6)	502	53 (10.3%)	449 (89.7%)	119	51 (42.9)	68 (57.1%)
	Geneva score (4–10)	683	95 (13.9%)	588 (86.1%)	119	37 (31.1%)	82 (68.9%)
High risk	Wells rule (>6)	216	56 (30.0%)	160 (70.0%)	22	14 (63.6%)	8 (36.4%)
	Geneva score (>10)	53	22 (41.3%)	31 (58.7%)	19	12 (63.2%)	7 (36.8%)

**Table:** (a) Relationship of CTPA and D-dimer in patients with a low-risk CDR

	Patients with low risk CDR		
		CTPA	
Wells rule (*N* = 32)	D-dimer	Positive	Negative
	Positive (*N* = 9)	2	7
	Negative (*N* = 23)	0	23
Geneva score (*N* = 35)			
	Positive (*N* = 8)	0	8
	Negative (*N* = 27)	0	27

**Table:** (b) Relationship of CTPA and D-dimer in patients with an intermediate-risk CDR

	Patients with intermediate risk CDR		
		CTPA	
Wells rule (*N* = 119)	D-dimer	Positive	Negative
	Positive (*N* = 51)	12	39
	Negative (*N* = 68)	4	64
Geneva score (*N* = 119)			
	Positive (*N* = 52)	19	39
	Negative (*N* = 67)	4	63
